# Atherogenic Burden and Remnant Cholesterol Associate With Electrophysiological Severity in Carpal Tunnel Syndrome

**DOI:** 10.1002/brb3.71637

**Published:** 2026-08-02

**Authors:** Aslı Aksoy Gündoğdu, Esen Çiçekli, Dilcan Kotan

**Affiliations:** ^1^ Department of Neurology Tekirdağ Namık Kemal University Faculty of Medicine Tekirdağ Türkiye; ^2^ Department of Neurology Sakarya University Faculty of Medicine Sakarya Türkiye

**Keywords:** atherogenic index of plasma, carpal tunnel syndrome, electrophysiology, remnant cholesterol

## Abstract

**Background:**

To investigate whether composite lipid indices and remnant cholesterol (RC) are associated with electrophysiological severity in carpal tunnel syndrome (CTS) and to evaluate their independent discriminative performance for advanced disease stage.

**Methods:**

In this cross‐sectional case‐control study, 124 patients with electrophysiologically confirmed CTS and 113 age‐ and sex‐matched controls were included. CTS severity was graded according to the Padua classification. Fasting lipid profiles were obtained, and composite atherogenic indices including the triglyceride‐to‐high‐density lipoprotein ratio (TG/HDL), the non‐HDL/HDL ratio, and the atherogenic index of plasma (AIP) and RC were calculated. Between‐group and severity‐based comparisons were performed. Multivariable logistic regression was used to assess independent associations with advanced CTS (moderate–severe vs. mild), and receiver operating characteristic (ROC) analyses evaluated discriminative performance.

**Results:**

CTS patients exhibited higher glucose, HbA1c, LDL‐cholesterol, total cholesterol, and RC levels than controls (all *p* < 0.05), whereas composite indices did not significantly differ between groups. Within the CTS cohort, lipid parameters, composite indices, and RC varied significantly across electrophysiological stages. In multivariable analysis adjusted for age and sex, non‐HDL/HDL ratio (OR = 2.99, 95% CI: 1.44–6.23), RC (OR = 3.12, 95% CI: 1.03–9.45), and AIP (OR = 0.27, 95% CI: 0.10–0.77) remained independently associated with advanced stage. The final model demonstrated moderate discriminative performance (area under the receiver operating characteristic curve [AUC] = 0.715, 95% CI: 0.619–0.811).

**Conclusion:**

Composite lipid indices and RC are independently associated with electrophysiological severity rather than CTS presence. These findings support a severity‐oriented metabolic framework in which systemic atherogenic burden may amplify median nerve dysfunction.

## Graphical abstract

Atherogenic burden was more strongly associated with electrophysiological severity than with CTS presence. Composite lipid indices and remnant cholesterol independently discriminated advanced CTS stages, suggesting that metabolic and microvascular factors may contribute to progressive median nerve dysfunction.

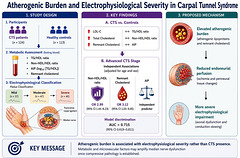

## 1 Introduction

Carpal tunnel syndrome (CTS) is the most common entrapment neuropathy of the upper limb and arises from compression of the median nerve within the confined osteofibrous canal (Padua et al. 2016). Although mechanical loading, hormonal factors, obesity, and systemic conditions are well‐recognized contributors, these factors do not fully explain individual susceptibility or the variability in electrophysiological severity (Padua et al. 2016; Lampainen et al. 2022; Shiri et al. 2015). Increasing evidence suggests that metabolic disturbances and microvascular dysfunction may play important roles in modulating median nerve vulnerability (Aslan et al. 2025; Genova et al. 2020; Ali et al. 2025; Acıman Demirel et al. 2021).

Peripheral nerves require a stable microvascular environment to sustain axonal integrity and Schwann cell function. Reductions in endoneurial perfusion may lead to ischemia and structural alterations in the perineural connective tissue (Saito et al. 2024; Wang et al. 2005; Lim et al. 2015). Elevated levels of atherogenic lipoproteins promote endothelial dysfunction and tissue remodeling, which may increase the likelihood of nerve compression and impair nerve conduction (Yeo et al. 2010; Savelieff et al. 2020; Ramírez‐Melo et al. 2025). Consistent with this concept, several clinical studies have reported higher triglyceride (TG), low‐density lipoprotein (LDL), and total cholesterol levels in individuals with CTS compared with healthy controls (Acıman Demirel et al. 2021; Yurdakul et al. 2015; Razavi et al. 2021; Hussein Awad El Gharieb et al. 2020).

Standard lipid parameters, however, may not capture the full spectrum of lipid‐related vascular risk. Composite lipid ratios such as the TG‐to‐high‐density lipoprotein (HDL) cholesterol ratio (TG/HDL), the non‐HDL to HDL ratio, and the atherogenic index of plasma (AIP = log10 [TG/HDL]) reflect endothelial stress, shifts toward small dense LDL particles, and increased atherogenic burden (Onat et al. 2010; Li et al. 2023; Li, Lu et al. 2025; Kırık et al. 2022). These indices have been widely used as markers of metabolic and microvascular injury in cardiometabolic disorders, yet their relevance to compression neuropathies has been explored only to a limited extent (Razavi et al. 2021; Onat et al. 2010; Kırık et al. 2022; Otelea et al. 2022). Previous CTS studies that relied primarily on conventional lipid profiles have yielded inconsistent results, with some studies reporting associations between dyslipidemia and nerve conduction abnormalities (Acıman Demirel et al. 2021; Yurdakul et al. 2015), whereas others observed no consistent relationship (Razavi et al. 2021; Hussein Awad El Gharieb et al. 2020).

In addition to composite lipid ratios, remnant cholesterol (RC), representing the cholesterol content of TG‐rich lipoprotein remnants, has emerged as an important marker of residual atherogenic risk (Valero et al. 2025). These particles can penetrate the vascular wall and contribute to endothelial dysfunction and microvascular injury (Borén et al. 2022). Despite growing evidence linking RC to cardiometabolic and microvascular pathology, its potential relevance to peripheral nerve dysfunction and entrapment neuropathies remains largely unexplored (Chen and Li 2023).

Importantly, emerging evidence indicates that metabolic and vascular factors may not uniformly influence the presence of CTS but may instead modulate the extent of median nerve dysfunction once compression has occurred (Acıman Demirel et al. 2021; Yurdakul et al. 2015). From this perspective, atherogenic burden may be closely related to electrophysiological severity than to disease presence alone (Acıman Demirel et al. 2021; Yurdakul et al. 2015). However, data addressing this severity‐oriented metabolic framework remain limited.

Despite growing evidence linking metabolic health to peripheral nerve function, the potential contribution of overall atherogenic burden in CTS remains insufficiently characterized (Savelieff et al. 2020; Razavi et al. 2021; Otelea et al. 2022). Few studies have simultaneously evaluated TG/HDL, non‐HDL/HDL, and AIP within a single cohort in relation to electrophysiological severity (Onat et al. 2010; Kırık et al. 2022).

Accordingly, the present study aims to compare TG/HDL, non‐HDL/HDL, and AIP values between individuals with CTS and healthy controls, and to evaluate how these indices are associated with electrophysiological severity and nerve conduction parameters. Understanding whether systemic atherogenic burden contributes to electrophysiological severity may provide insight into the metabolic modulation of peripheral nerve dysfunction.

## 2 Methods

### 2.1 Study Design and Participants

This cross‐sectional, case‐control study was conducted at our neurology clinic between October 2025 and January 2026. Consecutive adult patients aged 18–65 years with clinically and electrophysiologically confirmed CTS were recruited. An age‐ and sex‐matched control group was enrolled, consisting of healthy individuals without symptoms or clinical or electrophysiological findings suggestive of median nerve dysfunction.

Demographic and clinical information, including age, sex, body mass index (BMI), symptom duration, affected hand, and comorbidities, was recorded for all participants. Exclusion criteria included thyroid dysfunction, chronic kidney disease, rheumatologic or systemic inflammatory disease, peripheral neuropathy of any etiology, pregnancy, previous wrist trauma or surgery, and the current use of lipid‐lowering or glucose‐modifying medications. Individuals with incomplete laboratory or electrophysiological data were also excluded. Given the potential of extreme dietary regimens to alter lipid metabolism and atherogenic indices, individuals who had adhered to a ketogenic diet or a Dukan‐type high‐protein diet within the preceding 6 months were additionally excluded.

The study protocol was approved by the local institutional ethics committee (approval no: E‐43012747‐050.04‐543108‐660). All procedures were conducted in accordance with the Declaration of Helsinki. Written informed consent was obtained from all participants prior to inclusion in the study.

### 2.2 Diagnostic Criteria and Electrophysiological Assessment

The diagnosis of CTS was established based on typical clinical features, including paresthesia, pain, or numbness in the median nerve distribution, and was confirmed by nerve conduction studies. All electrophysiological examinations were performed using standard surface electrode techniques with skin temperature maintained above 32°C.

Median and ulnar nerve sensory and motor conduction parameters were evaluated in accordance with established protocols. Median nerve sensory and motor distal latencies, sensory and motor conduction velocities, compound muscle action potentials (CMAPs), and sensory nerve action potentials (SNAPs) were recorded.

CTS severity was graded using the Padua classification, which integrates sensory and motor conduction abnormalities to categorize CTS as mild, moderate, or severe.

### 2.3 Biochemical Measurements

Fasting venous blood samples were obtained after at least 8 h of overnight fasting. Serum concentrations of total cholesterol, HDL, LDL, and TG were measured using standardized enzymatic assays on an automated analyzer.

Atherogenic lipid indices were calculated as follows:

- TG/HDL ratio = TG / HDL cholesterol
- Non‐HDL/HDL ratio = (Total cholesterol − HDL cholesterol) / HDL cholesterol
- AIP = log10(TG / HDL cholesterol)
- RC = Total cholesterol − HDL cholesterol − LDL cholesterol

All biochemical analyses were performed in the same certified laboratory to ensure analytical consistency.

### 2.4 Statistical Analysis

#### 2.4.1 Sample Size Estimation

A sample size calculation was performed prior to study initiation using G*Power software (version 3.1; Heinrich‐Heine‐Universität Düsseldorf, Germany). For a two‐sided comparison between two independent groups (CTS patients and healthy controls), corresponding to the primary study objective, an alpha level of 0.05, a statistical power (1 − β) of 0.80, and a moderate effect size (Cohen's *d* = 0.50) were assumed. Based on these parameters, the minimum required total sample size was 128 participants (64 per group). The larger final sample size allowed for more robust subgroup and severity‐based analyses.

Statistical analyses were performed using IBM SPSS Statistics (version 21). The distribution of continuous variables was assessed using the Kolmogorov–Smirnov test. Continuous variables are presented as mean ± standard deviation for normally distributed data and as median (interquartile range) for non‐normally distributed data. Categorical variables are expressed as a number (percentage).

Comparisons between CTS patients and controls were conducted using the independent samples *t*‐test for normally distributed variables and the Mann–Whitney *U* test for non‐normally distributed variables, as appropriate. Differences among CTS severity groups were evaluated using the Kruskal–Wallis test, followed by Bonferroni‐corrected post hoc pairwise comparisons when significant. For severity‐based analyses, CTS was additionally dichotomized to enable stage discrimination modeling.

To evaluate the discriminative capacity of composite lipid indices across electrophysiological stages, binary logistic regression analyses were performed, comparing severe with mild–moderate CTS and mild with moderate–severe CTS. The model demonstrating superior discriminative performance (higher AUC) and better overall fit (lower −2 log likelihood) was selected for final multivariable analysis. Continuous predictors were standardized (*z*‐scores) to facilitate comparison of effect sizes, and age and sex were included as covariates. Multicollinearity was assessed using variance inflation factors (VIF), with values < 5 considered acceptable; variables demonstrating excessive collinearity were not entered simultaneously into multivariable models. Receiver operating characteristic (ROC) curve analyses were conducted to assess the discriminative performance of individual composite indices and the final multivariable model. Optimal cut‐off values were determined using the Youden index.

All statistical tests were two‐tailed, and a *p* value < 0.05 was considered statistically significant.

## 3 Results

### 3.1 Study Population and Baseline Characteristics

A total of 237 individuals were included in the study, comprising 124 patients with electrophysiologically confirmed CTS and 113 age‐ and sex‐matched healthy controls. Demographic characteristics were comparable between groups (Table 1). There were no significant differences in age (*p* = 0.098) or sex distribution (*p* = 0.664). Compared with controls, patients with CTS exhibited significantly higher fasting glucose, HbA1c, LDL‐cholesterol, total cholesterol, and RC levels (all *p* < 0.05). In contrast, TG, TG/HDL ratio, AIP, and non‐HDL/HDL ratio did not differ significantly between groups.

**TABLE 1:** Demographic and baseline biochemical characteristics of patients with carpal tunnel syndrome and healthy controls.

Variable	CTS (*n* = 124)	Controls (*n* = 113)	*p* value
Age, years (mean ± SD)	46.3 ± 8.6	44.0 ± 12.4	0.098
Female sex, *n* (%)	92 (74.2)	81 (71.7)	0.664
Fasting glucose, mg/dL	98 (20.3)	93 (13.3)	0.001
HbA1c, %	5.7 (0.1)	5.5 (0.3)	< 0.001
HDL‐cholesterol, mg/dL	53 (2.0)	45 (14.0)	< 0.001
LDL‐cholesterol, mg/dL	116.6 (16.9)	100 (31.9)	< 0.001
Total cholesterol, mg/dL	201 (17.0)	175 (40.0)	< 0.001
Triglycerides, mg/dL	117 (11.5)	139 (10.2)	0.293
Remnant cholesterol, mg/dL (RC)	30 (9.0)	26 (20.5)	0.029
Atherogenic index of plasma (AIP)	0.35 (0.06)	0.44 (0.44)	0.100
TG/HDL ratio	2.21 (0.35)	2.76 (2.81)	0.100
Non‐HDL/HDL ratio	2.78 (0.45)	2.72 (1.12)	0.225

*Note*: Values are presented as mean ± standard deviation (SD) for normally distributed variables and median (interquartile range [IQR]) for non‐normally distributed variables, and number (percentage) for categorical variables. Between‐group comparisons were performed using Student's *t*‐test or Mann–Whitney *U* test as appropriate.

### 3.2 Biochemical Parameters According to Electrophysiological Severity

When CTS patients were stratified according to electrophysiological severity (mild, moderate, severe), several lipid‐related parameters demonstrated significant between‐group differences (Table 2). LDL‐cholesterol and total cholesterol levels progressively increased with advancing severity (both *p* ≤ 0.001). Significant between‐group differences were also observed for TG, RC, AIP, TG/HDL ratio, and non‐HDL/HDL ratio (all *p* < 0.05).

**TABLE 2:** Biochemical parameters according to electrophysiological severity of CTS.

Variable	Mild CTS (*n* = 37)	Moderate CTS (*n* = 47)	Severe CTS (*n* = 40)	*p* value
Fasting glucose, mg/dL	98.5 (22.5)	96 (20)	99 (16)	0.202
HbA1c, %	5.65 (0.10)	5.70 (0.10)	5.60 (0.10)	< 0.001
HDL‐cholesterol, mg/dL	53.0 (1)	52.0 (3)	54.0 (2)	< 0.001
LDL‐cholesterol, mg/dL	107.0 (11.5)	116.6 (12.4)	120.9 (6.5)	< 0.001
Total cholesterol, mg/dL	190.0 (6)	201.0 (10)	204.0 (7)	0.001
Triglycerides, mg/dL	117.0 (1)	115.0 (13)	121.0 (24)	0.010
Remnant cholesterol, mg/dL (RC)	30.0 (6.4)	32.4 (10)	29.1 (4.7)	0.023
Atherogenic index of plasma (AIP)	0.34 (0.01)	0.35 (0.10)	0.35 (0.06)	0.004
TG/HDL ratio	2.24 (0.33)	2.87 (0.71)	2.78 (0.15)	0.028
Non‐HDL/HDL ratio	2.58 (0.37)	3.10 (0.78)	2.80 (0.46)	< 0.001

*Note*: Values are presented as median (interquartile range [IQR]). Comparisons across CTS severity groups were performed using the Kruskal–Wallis test with Bonferroni‐corrected post hoc analyses.

Abbreviations: AIP, atherogenic index of plasma; CTS, carpal tunnel syndrome; RC, remnant cholesterol.

Post hoc analyses demonstrated that LDL‐cholesterol, total cholesterol, AIP, and non‐HDL/HDL ratio were significantly higher in moderate and severe CTS compared with mild cases, whereas TG levels were primarily elevated in severe CTS relative to mild disease. RC showed variability across stages but without a consistent stepwise increase. Overall, these findings indicate a stage‐dependent alteration in atherogenic lipid burden and provide the rationale for subsequent multivariable analyses aimed at evaluating the discriminative capacity of composite lipid indices.

### 3.3 Multivariable Logistic Regression Analysis

**FIGURE 1:**
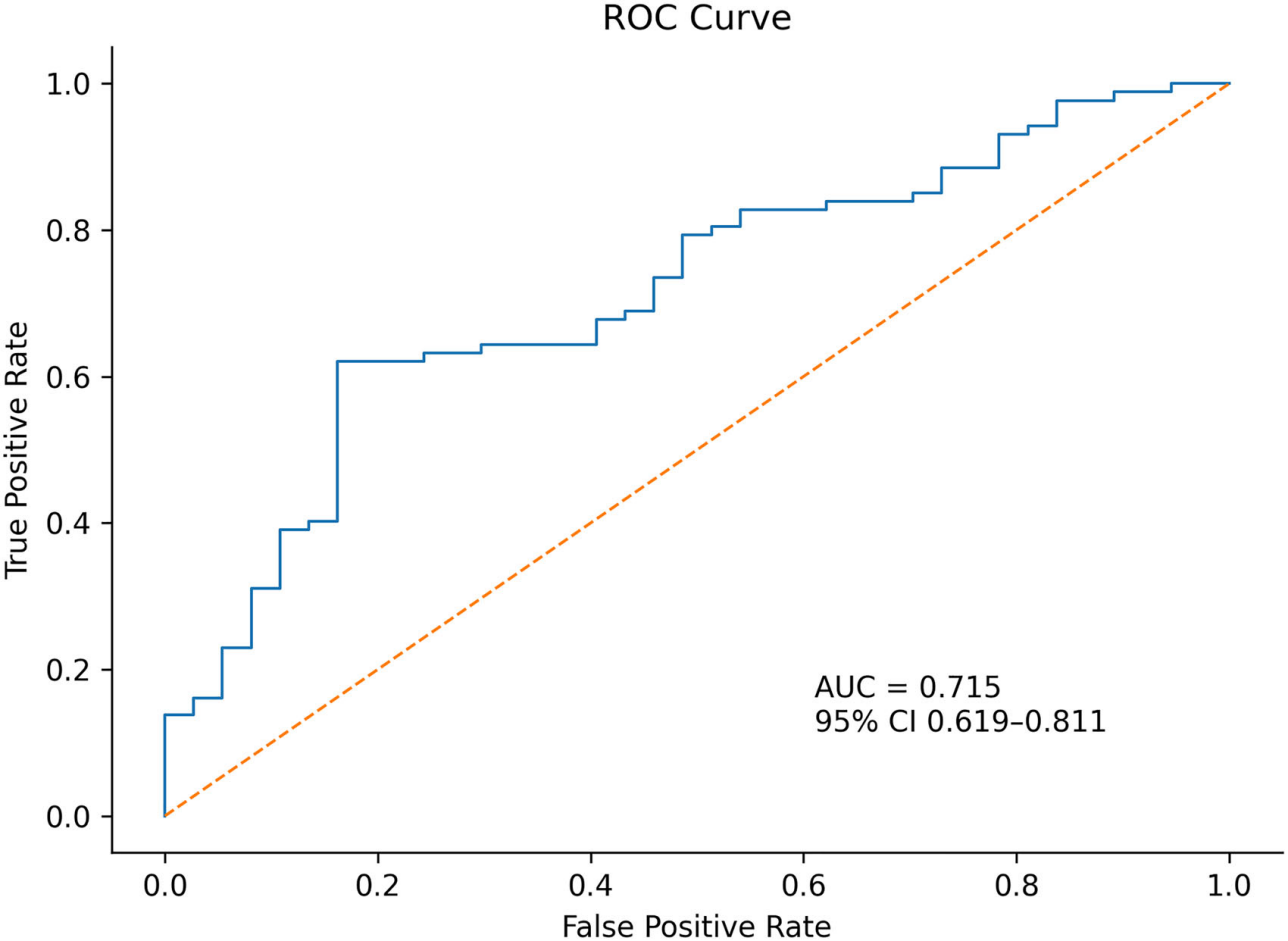
ROC curve demonstrating the discriminative performance of the multivariable model for advanced CTS (moderate–severe vs. mild). AUC = 0.715 (95% CI: 0.619–0.811). AIP, atherogenic index of plasma; RC, remnant cholesterol.

Binary logistic regression was performed to evaluate the independent association of composite lipid indices with advanced electrophysiological stage (moderate–severe vs. mild) (Table 3). All continuous predictors were standardized (*z*‐scores) prior to modeling to allow comparison of effect sizes. After adjustment for age and sex, the non‐HDL/HDL ratio remained independently associated with advanced stage (OR = 2.99, 95% CI: 1.44–6.23; *p* = 0.003). RC was also independently associated with advanced electrophysiological stage (OR = 3.12, 95% CI: 1.03–9.45; *p* = 0.044). Similarly, AIP remained significantly associated after multivariable adjustment (OR = 0.27, 95% CI: 0.10–0.77; *p* = 0.014). In contrast, age and sex were not independently associated with stage discrimination. All predictors in the final model demonstrated acceptable collinearity levels (all VIF < 5). The model demonstrated moderate discrimination with an area under the receiver operating characteristic curve (AUC) of 0.715 (95% CI: 0.619–0.811) (Figure 1). Results remained materially unchanged after additional adjustment for BMI and HbA1c in sensitivity analyses, with the associations of non‐HDL/HDL ratio, AIP, and RC with advanced CTS stage remaining statistically significant.

**TABLE 3:** Multivariable logistic regression for advanced electrophysiological stage (moderate–severe vs. mild CTS).

Predictor	OR (95% CI)	*p* value
Age	1.04 (0.99–1.09)	0.170
Sex	0.91 (0.36–2.31)	0.842
Remnant cholesterol (RC)	3.12 (1.03–9.45)	0.044
Atherogenic index of plasma (AIP)	0.27 (0.10–0.77)	0.014
Non‐HDL/HDL ratio	2.99 (1.44–6.23)	0.003

*Note*: Composite lipid indices were standardized (*z*‐scores); odds ratios represent the effect per 1 SD increase. Age was entered as a continuous covariate and sex as a binary covariate. Model performance: AUC = 0.715 (95% CI: 0.619–0.811); Nagelkerke *R* ^2^ = 0.171; Hosmer–Lemeshow *p* = 0.263.

### 3.4 ROC Analysis of Individual Composite Indices

ROC analyses further assessed the ability of individual composite indices to discriminate advanced (moderate–severe) from mild CTS (Table 4). AIP demonstrated the highest discriminative performance (AUC = 0.685), followed closely by non‐HDL/HDL ratio (AUC = 0.672). RC showed lower discriminative capacity (AUC = 0.571). The optimal cut‐off for AIP was 0.345, yielding 79% sensitivity and 78% specificity. The relatively modest univariate ROC performance of RC contrasts with its independent association in multivariable modeling, underscoring that discrimination and adjusted association capture different statistical properties.

**TABLE 4:** ROC analysis of individual composite lipid indices for discriminating advanced CTS.

Variable	AUC (95% CI)	Optimal cut‐off	Sensitivity	Specificity
Remnant cholesterol (RC)	0.571 (0.468–0.674)	30.2	0.46	0.89
Atherogenic index of plasma (AIP)	0.685 (0.588–0.782)	0.345	0.79	0.78
Non‐HDL/HDL ratio	0.672 (0.574–0.770)	2.62	0.79	0.73

*Note*: Optimal cut‐off values were determined using the Youden index.

Abbreviation: AUC, area under the receiver operating characteristic curve.

## 4 Discussion

Our findings address an important gap in the understanding of metabolic contributions to CTS by demonstrating that atherogenic burden aligns more closely with electrophysiological stage discrimination than with disease presence alone. By integrating conventional lipid parameters, composite lipid indices, and RC, this study provides evidence that lipid‐related metabolic factors are more strongly associated with disease severity than with the mere occurrence of CTS. These observations suggest that once compressive pathology is established, systemic atherogenic mechanisms may modulate intraneural vulnerability and contribute to more advanced electrophysiological impairment.

In line with previous observations, our cohort demonstrated higher fasting glucose, HbA1c, LDL‐cholesterol, total cholesterol, and RC levels compared with healthy controls (Yurdakul et al. 2015; Hussein Awad El Gharieb et al. 2020). Metabolic abnormalities, including dyslipidemia and impaired glucose metabolism, are more prevalent among individuals with CTS, supporting a contributory role of systemic metabolic stress in disease susceptibility (Acıman Demirel et al. 2021; Yurdakul et al. 2015). Although HbA1c values differed statistically across CTS severity groups, the absolute differences were small and unlikely to reflect clinically meaningful alterations in glycemic control. Importantly, studies incorporating electrophysiological grading have reported higher LDL‐cholesterol and total cholesterol levels in patients with more advanced CTS, suggesting that metabolic alterations become more evident when disease severity is taken into consideration (Acıman Demirel et al. 2021).

However, findings across the literature remain heterogeneous. Investigations focusing solely on case‐control comparisons without severity stratification have often reported no significant differences in conventional lipid parameters between CTS patients and controls (Razavi et al. 2021; Hussein Awad El Gharieb et al. 2020), whereas others have linked elevated LDL‐cholesterol and total cholesterol levels to CTS prevalence, particularly in populations with metabolic comorbidities (Acıman Demirel et al. 2021; Yurdakul et al. 2015; Hussein Awad El Gharieb et al. 2020). Our findings help reconcile these divergent observations by suggesting that metabolic relevance becomes clearer when electrophysiological severity is considered rather than binary disease presence.

In contrast to conventional lipid parameters, composite lipid indices, including TG/HDL, non‐HDL/HDL, and the AIP, did not distinguish CTS patients from controls. This finding aligns with previous studies reporting null associations between nontraditional lipid ratios and CTS presence, particularly in heterogeneous cohorts without electrophysiological severity stratification (Razavi et al. 2021; Uzkeser et al. 2011). Together, these observations suggest that composite lipid indices may not function as binary risk markers of CTS susceptibility. Rather, they may reflect cumulative metabolic and vascular stress, which becomes clinically relevant in the context of established nerve compression (Assempoor et al. 2025; Afsin et al. 2021).

When CTS patients were stratified according to the Padua electrophysiological severity grades, a clear and clinically relevant pattern emerged, with LDL‐cholesterol, total cholesterol, TG, composite lipid indices, and RC varying significantly across severity categories. These findings are consistent with previous reports demonstrating associations between conventional serum lipid parameters and electrophysiological severity of CTS (Razavi et al. 2021). Importantly, our results extend this evidence by showing that composite lipid indices and RC also exhibit severity‐dependent patterns.

Moreover, in multivariable modeling, non‐HDL/HDL ratio and RC remained independently associated with advanced stage after adjustment for age and sex, and AIP also demonstrated independent association. The inverse association observed for AIP in the multivariable model likely reflects collinearity and shared variance among lipid‐related indices within the multivariable model (AIP, non‐HDL/HDL ratio, and RC), which capture overlapping aspects of atherogenic burden. When examined in separate models, AIP demonstrated a positive association with advanced stage, suggesting that the inverse direction observed in the multivariable model reflects statistical interaction among correlated predictors rather than a biologically protective effect. The final model showed moderate discrimination, supporting the notion that composite lipid indices contribute to stage differentiation beyond simple disease presence (Yang et al. 2023). This level of accuracy is consistent with multifactorial conditions such as CTS, in which metabolic factors interact with mechanical and anatomical determinants.

Studies that failed to detect associations between lipid parameters and CTS often relied on case‐control comparisons without standardized electrophysiological grading (Razavi et al. 2021; Hussein Awad El Gharieb et al. 2020), which may partly explain the heterogeneity across the literature. In this context, our findings support a stage‐oriented metabolic model of CTS, in which systemic atherogenic burden may amplify the functional impact of mechanical compression within the carpal tunnel. Accordingly, a severity‐oriented metabolic framework suggests that metabolic and vascular factors may not primarily determine whether CTS develops, but rather modulate the degree of neural vulnerability once compressive pathology is established. Systemic atherogenic burden may therefore amplify microvascular stress, impair intraneural perfusion, and increase susceptibility of the median nerve to mechanical compression, thereby contributing to more advanced electrophysiological impairment (Iqbal et al. 2021; Horton and Barrett 2020; Maiuolo et al. 2019).

From a biological perspective, the observed association between atherogenic burden and CTS severity is supported by established neurovascular mechanisms. Peripheral nerves depend on an intact microvascular supply, known as the vasa nervorum, to maintain axonal integrity and Schwann cell function (Iqbal et al. 2021). Dyslipidemia‐related endothelial dysfunction may impair microvascular vasoreactivity and tissue perfusion, leading to reduced oxygen and nutrient delivery at the endoneurial level (Horton and Barrett 2020). In peripheral nerves, such microvascular alterations can increase susceptibility to ischemic stress and structural changes within the perineural connective tissue (Maiuolo et al. 2019). Within the restricted anatomical space of the carpal tunnel, even subtle microvascular compromise may amplify the electrophysiological consequences of mechanical compression. These mechanisms provide a biologically plausible explanation for why systemic atherogenic burden may contribute to more advanced electrophysiological impairment in CTS. Composite lipid indices, which integrate TG enrichment, HDL deficiency, and a predominance of small dense LDL particles, may therefore provide a more comprehensive representation of cumulative vascular stress than isolated lipid measures, offering a biologically plausible link between systemic atherogenic burden and advanced median nerve dysfunction (Assempoor et al. 2025; Rabiee Rad et al. 2024).

A distinctive aspect of this study is the evaluation of RC within a severity‐stratified CTS cohort (Acıman Demirel et al. 2021; Razavi et al. 2021). Although RC levels varied across electrophysiological severity groups, a strictly stepwise increase was not observed in the descriptive comparisons. However, multivariable regression analysis evaluates the independent contribution of RC after adjustment for other covariates. Therefore, RC may contribute to stage discrimination when considered within the broader metabolic profile, even if the unadjusted distribution across severity groups is not strictly monotonic. Remnant lipoproteins readily penetrate the vascular wall and promote endothelial dysfunction and low‐grade inflammation (Baratta et al. 2023; Pintó et al. 2023). While RC has been extensively studied in cardiovascular and metabolic disorders, data addressing its role in peripheral nerve dysfunction remain limited (Li, Li, and Wu et al. 2025). These findings extend this concept of residual atherogenic risk to entrapment neuropathies and suggest that RC may serve as a marker of CTS severity rather than disease susceptibility.

Sex‐based analyses revealed no significant differences in electrophysiological severity distribution or metabolic parameters among CTS patients. These findings are consistent with prior studies reporting comparable electrophysiological severity between male and female patients and suggest that the observed metabolic associations are unlikely to be driven by sex‐related confounding (Cazares‐Manríquez et al. 2020; Eros et al. 2024; Mathew and John 2021).

The present findings have potential clinical implications. Because composite lipid indices and RC can be derived from routine laboratory testing, they may serve as accessible adjunctive markers for identifying patients at risk for more severe CTS. Rather than functioning as diagnostic tools, these indices may be particularly relevant for severity stratification and for identifying individuals who may benefit from closer clinical monitoring or optimization of metabolic risk factors. These findings do not imply causality but highlight the potential relevance of metabolic optimization in patients presenting with advanced CTS. From a research perspective, these findings underscore the need for longitudinal studies integrating metabolic, vascular, and neurophysiological assessments to clarify temporal relationships and underlying biological mechanisms.

Several methodological features strengthen the validity of these findings, including the integration of conventional lipid parameters, multiple composite indices, and RC within a severity‐stratified electrophysiological framework, combined with multivariable modeling. Nevertheless, certain limitations should be acknowledged. The cross‐sectional design precludes causal inference and does not allow determination of whether atherogenic burden precedes electrophysiological progression in CTS. Direct assessments of microvascular function were not performed, limiting mechanistic interpretation. Although individuals receiving lipid‐lowering therapies were excluded, several other potential confounding factors were not specifically evaluated in the present study. In particular, occupational exposure, repetitive wrist movements, symptom duration, bilateral involvement, and lifestyle factors such as dietary habits and physical activity may influence CTS severity and metabolic parameters. Therefore, residual confounding cannot be entirely excluded, and future prospective studies incorporating detailed occupational and lifestyle assessments are warranted. In addition, electrophysiological severity was assessed at a single time point, preventing evaluation of temporal changes in median nerve function. Finally, the modest discriminative performance of the model suggests that lipid‐related factors represent only one component of the multifactorial pathophysiology of CTS.

## 5 Conclusion

In summary, lipid‐related metabolic burden, particularly when assessed using composite lipid indices and RC, appears to relate more closely to electrophysiological severity than to the presence of CTS. These findings support a severity‐oriented metabolic framework in which systemic atherogenic mechanisms may amplify the functional consequences of mechanical compression in CTS.

## Author Contributions

**Aslı Aksoy Gündoğdu**: conceptualization, methodology, formal analysis, writing – original draft, supervision. **Esen Çiçekli**: data curation, investigation, writing – review and editing. **Dilcan Kotan**: investigation, resources, writing – review and editing.

## Funding

The authors have nothing to report.

## Ethics Statement

The study was approved by the local institutional ethics committee (approval no: E‐43012747‐050.04‐543108‐660). All procedures were conducted in accordance with the Declaration of Helsinki. Written informed consent was obtained from all participants.

## Conflicts of Interest

The authors declare no conflicts of interest.

## Data Availability

The data supporting the findings of this study are available from the corresponding author upon reasonable request.
